# Area-level HIV risk and socioeconomic factors associated with willingness to use PrEP among Black people in the U.S. South

**DOI:** 10.1016/j.annepidem.2019.11.002

**Published:** 2019-11-30

**Authors:** Yusuf Ransome, Laura M. Bogart, Ichiro Kawachi, Anna Kaplan, Kenneth H. Mayer, Bisola Ojikutu

**Affiliations:** a Department of Social and Behavioral Sciences, Yale School of Public Health, New Haven, CT; b Pardee RAND Graduate School, RAND Corporation, Santa Monica, CA; c Department of Social and Behavioral Sciences, Harvard T.H. Chan School of Public Health, Boston, MA; d Cambridge Public Health Department, Cambridge, MA; e The Fenway Institute, Fenway Health, Boston, MA; f Department of Medicine, Beth Israel Deaconess Medical Center, Boston, MA; g Harvard Medical School, Boston, MA; h Brigham and Women's Hospital, Boston, MA

**Keywords:** HIV, Black/African Americans, Pre-exposure prophylaxis (PrEP), South, United States (U.S.)

## Abstract

**Purpose:**

In the United States (U.S.), southern states have the highest HIV incidence. Uptake of pre-exposure prophylaxis (PrEP) has been slow among Black people, particularly in the South. We know little about how area-level HIV risk influences one's willingness to use PrEP.

**Methods:**

169 Black participants across 142 ZIP codes in the South completed the 2016 National Survey on HIV in the Black Community. We performed log-binomial regression to estimate the prevalence risk associated with residing in the upper 25th percentile of increases in new HIV diagnosis (2014–2015) within ZIP code and an individual's willingness to use PrEP, adjusting for individual and area-level covariates.

**Results:**

Participants were 68% female, mean age of 36 years, and 24% willing to use PrEP. Among the ZIP codes, 23% were within Atlanta, GA. The median increase in new HIV diagnoses was 25 per 100,000 population from 2014 to 2015 (IQR, 14–49). Participants living in ZIP codes within the upper 25th (compared-to-lower 75th) percentile of new HIV diagnoses were more willing to use PrEP (adjusted prevalence ratio (aPR) = 2.02, 95% CI = 1.06–3.86, *P* = .03). Area-level socioeconomic factors attenuated that association (aPR = 1.63, 95% CI = 0.78–3.39, *P* = .19).

**Conclusions:**

Area-level factors may influence PrEP uptake among Black people in the South.

## Introduction

Pre-exposure prophylaxis (PrEP) is a highly effective means of preventing HIV infection [1]. The United States (U.S.) Food and Drug Administration approved a once-daily pill (tenofovir/emtricitabine) for PrEP in 2012 [2]. Evidence from clinical studies showed that PrEP has significantly reduced HIV incidence rates in heterosexually discordant as well as same-sex discordant couples [3, 4]. Uptake of PrEP in the U.S. general population is low; approximately 8% of eligible persons are actively using PrEP [2] although higher among men who have sex with men (MSM) at 35% [5]. Significant racial, ethnic, and geographic inequalities in access to and usage of PrEP also exist [5].

Among at-risk U.S. Black/African Americans (hereafter Black people), PrEP use is particularly low compared to people of other race and ethnicities [6]. Recent data indicate that although Black men and women accounted for a higher proportion of persons with PrEP indicators (i.e., eligible for PrEP) (44% vs. 26%), they received PrEP at nearly five times a lower rate compared with white women and men (13% vs. 69%) [7]. According to a 2018 Centers for Disease Control and Prevention (CDC) report, only 1% of eligible Black people were prescribed PrEP [2]. To date, the social determinants of low PrEP uptake among Black people have not been well described. Social determinants of health (SDOH) focus on root causes of disease and behavior [8], which are often broader social factors (e.g., education, inequality, social cohesion), and SDOH can operate at multiple levels (e.g., individual or interpersonal) and across multiple environments such as neighborhood of residence or workplace [8, 9]. Part of the reason for the paucity of SDOH studies in PrEP willingness and uptake is because determining which of the numerous SDOH factors to study and intervene on is complex and dependent on the health or behavioral issue, target audience (e.g., adolescents, immigrants, Black people) and whether one has an emic perspective of the situation if they are part of the target group [10].

When considering geography, PrEP uptake is lowest in the U.S. South. Despite being home to more than 50% of newly diagnosed individuals in 2016, population estimates from the end of Quarter 4 in 2017 noted that the rate of PrEP use in the South was 21.0 per 100,000 compared to 43.7 per 100,000 in the northeast [11]. Moreover, only 25% of clinics prescribing PrEP were in the South. For example, the rate of clinics prescribing PrEP in Birmingham and Atlanta (19.0 and 14.5, respectively, per 1000 new HIV diagnoses) is significantly lower than the rate of clinics in northeast cities such as Philadelphia and New York (58.8 and 58.0, respectively, per 1000 new HIV diagnoses) [12].

Race/ethnicity and geography are often not independent risk factors, but rather intersect to widen HIV disparities [13]. To that extent, the HIV burden among Black people is most pronounced in the U.S. South. Sixty-three percent of new HIV diagnoses in 2016 and 58% of Black people living with HIV are in the South [14]. Three of five states that accounted for 35% of PrEP need among Black MSM are in the South (Florida, Georgia, Texas) [15]. In this study, we investigated ecological-level SDOH of willingness to use PrEP among a sample of Black people in the U.S. South.

We focus on SDOH at the ecological level for several reasons. First, the current body of work on PrEP uptake has focused mainly on sociodemographic, behavioral, or psychosocial factors associated with PrEP willingness, but such factors are necessarily unique to Black people or the U.S. South. For instance, one recent study identified that among Black people, single relationship status, depression, incarceration history, doctor visits, PrEP knowledge, and HIV conspiracy beliefs were all significantly associated with willingness to use PrEP [16]. Another recent study, among a sample of Black people, found that religious factors such as exposure to HIV messages by faith leaders were associated with willingness to use PrEP [17]. Other studies on PrEP uptake among Black MSM studied individual-level psychosocial factors such as PrEP stigma, PrEP conspiracy beliefs, and PrEP interest [18, 19].

Other studies that include racially and geographically diverse populations found that interpersonal factors such as social stigma among potential PrEP users influenced one's likelihood of using PrEP [20]. Health-care system factors prohibiting PrEP uptake include physicians’ racial stereotype bias [21] and stigma [22], medical mistrust [23], and lack of adequate knowledge or training to counsel patients eligible for PrEP [24]. Structural factors influencing willingness and usage of PrEP include the high out-of-pocket cost for the prescriptions [25] and variation in the density of clinics that prescribe PrEP [26].

Beyond those aforementioned factors, there are also SDOH at the area/community-level HIV transmission factors such as HIV incidence and prevalence and viral load that may impact HIV risk and prevention variables among Black people [27]. In this study, the primary area-level SDOH variable is the prevalence rate of new HIV diagnosis in a ZIP code, which we operationalize as *area-level HIV risk*. That exposure qualifies as an SDOH because there are vast differences in exposure, vulnerability, and consequences for some groups of people [8, 10]. The annual number of new HIV diagnoses is the closest reasonable proxy for populations that need to use PrEP [11] as well as HIV incidence [15, 28]. However, incidence data are unavailable at the population level [29].

New HIV diagnosis is also a reasonable proxy for area-level HIV risk among Black people. It is well known that high HIV prevalence in dense sexual networks is one primary ecological factor that interacts with social network factors to heighten HIV risk among this population [30-32]. Finally, the CDC recommends that physicians consider the epidemiologic context such as living in an area with high HIV prevalence, which is included in the summary of guidance for PrEP among heterosexual men and women [33]. However, in practice, there is no evidence on the percentage of clinicians that actually use this criterion to prescribe PrEP.

In this study, we hypothesize that participants living in places with higher area-level HIV risk will have higher likelihood of willingness to use PrEP, compared to those who live in areas with lower HIV risk. Based on diffusion of innovation and information theory as applied to HIV [34], people who live in places with higher HIV prevalence may be more aware of their HIV risk. Awareness and dialogue [35] about HIV may be facilitated by social media stories, whether true or false [36], or through other facts-based online platforms such as AIDSvu [37].

Psychosocial determinants such as stigma are other potential pathways through which area-level factors may influence individuals’ willingness to use PrEP. For instance, individuals may be less likely to internalize any societal-level stigma associated with HIV if the discourse surrounding HIV risk is focused on an ecological definition (e.g., high area-level HIV prevalence) in contrast to an *individual* definition of risk (e.g., an at-risk person being an injection drug user or participating in condomless anal intercourse).

The secondary aim of this paper was to examine whether more common SDOH factors mitigate any positive associations between area-level HIV risk and PrEP willingness. Friedman and colleagues presented a theoretical model identifying the social and structural risk of HIV among Black people [38]. The model postulates that high HIV rates among this population in the United States are a function of racialized factors—factors rooted racial discriminatory foundations such as “racialized economy,” which includes socioeconomic deprivation [38]. Thus, we expect that deprivation will account for some of the variance in the association. Therefore, our second hypothesis is that socioeconomic factors such as area-level deprivation and income inequality will erode some of any positive association between area-level HIV risk and individual's willingness to use PrEP.

Lastly, in this study, we used data for general population, including those self-identified at high HIV risk. We draw on Geoffrey Rose's theories in preventive epidemiology, specifically the *Population-level Strategy* [39] for why such an approach is justified. Rose demonstrated that for some health outcomes, focusing solely on high-risk individuals is an inadequate approach. Two of several weaknesses of the individual-level high-risk approach as identified by Rose includes: (1) poor ability to predict the future of who will be at risk, and that (2) one's behavior is structured by larger social norms. Rose postulates that for some outcomes, a larger number of people at modest risk may give rise to more cases of disease than a small number of high-risk individuals [39]. Therefore, understanding the association between ecological-level HIV risk among the general population, as we do in this study, can provide evidence needed to shift the entire distribution of all who are exposed to HIV now and who may be as definitions of HIV risk may change. To date, there is no consensus on what is defined as HIV risk, because it is socially constructed [40], and therefore problematic because it impedes prevention efforts. PrEP uptake may be low because people resist the cultural labels constructed as “high risk” [41]. One recent experimental study showed that people were less willing to support policies for making PrEP affordable when it was for Black gay men compared to the general population [42]. Others recommend a routine approach to sharing information about PrEP to the wider community under the premise that people within one's social network may help disseminate PrEP knowledge and reduce barriers such as provider mistrust [43].

Our premise then is that a population strategy can support interventions directed toward addressing the social and normative environmental determinants of risk as well as individual risk behaviors. We therefore tested whether higher area-level HIV risk (i.e., rate of new HIV diagnoses in a ZIP code) is associated with higher willingness to use PrEP (AIM 1) and whether including area-level socioeconomic factors (e.g., deprivation and income inequality) attenuates that association (AIM 2).

## Methods

### Data

Data were drawn from the 2016 National Survey on HIV in the Black Community (NSHBC), which is a cross-sectional survey of Black people in the United States. The NSHBC asked questions about cultural, structural, and psychosocial drivers of racial/ethnic disparities in the HIV epidemic such as homophobia, religious involvement, racial discrimination, and willingness to use PrEP. Eligible participants were between the ages of 18 and 50, self-identified Black race, and living in households throughout the United States. The sample was drawn from a probability-based Web panel (KnowledgePanel®) that is representative of adults living within U.S. households. Individuals were provided with Internet access and a computer, if needed, and respondents completed the survey online. People from institutionalized settings or homeless or transiently house were not sampled. All 1969 Black/African American participants in the panel were sampled and 46% of those people (*n* = 898) consented to complete a brief sociodemographic survey confirming their race and age. Of those who consented, 89% of respondents (*n* = 868/970) were eligible to participate in NSHBC and fully completed the survey. Written consent was obtained from participants before they took the survey. The survey was written at a Flesch–Kincaid grade level of 3.6, which corresponds to a fourth-grade reading level. A detailed description of survey sampling, design, and administration has been published previously [44]. Respondent ZIP codes corresponding to their place of reported residence were collected directly from the survey company. All study protocols were approved by the Boston Children's Hospital Institutional Review Board.

### Participants

Analyses were restricted to participants whose self-reported residence, which we verified through their ZIP code (*n* = 437), was in cities in the U.S. South, as defined by the U.S. Census Bureau [45]. Some cities included Dallas, TX, and Atlanta, GA. We further restricted the sample to participants living in a ZIP code that could be linked to new HIV diagnosis data for the years 2014 and 2015 and excluded people living with HIV (*n* = 2) since they are not eligible for PrEP. The final analytic sample included 169 out of 437 persons (38.7%) who reported residence in the U.S. South.

### Informed consent and ethics

Written consent was obtained from participants before they took the survey, which was one of the first notifications that appeared on the screen before. If they did not consent, the study was terminated. The study procedures were approved by Boston Children's Hospital IRB Ethics Committee.

### Measures

#### Area-level HIV risk

We obtained new HIV diagnoses data from AIDSVu.org for years 2014 and 2015 at the ZIP-code level [37]. AIDSvu ZIP-code level data reflects the most known recent address for the person living with HIV. We calculated the change in new HIV diagnosis rate per 100,000 population as the difference in rates between the years. We then classified the change score into the upper 25th percentile compared to the bottom 75th percentile. ZlP codes within the upper 25th percentile represented high area-level HIV risk.

#### Area-level socioeconomic factors

We examined the following area-level socioeconomic factors known to be associated with HlV-related transmission and prevention: socioeconomic deprivation (SED) and income inequality [29, 46, 47]. The first measure, SED was an index created from a principal component analysis of the following variables: proportion of persons living below the Federal poverty level, the proportion of persons unemployed, and median household income. These variables yielded a Cronbach's alpha of 0.88 in this study sample. The second socioeconomic factor is income inequality assessed through the GINI coefficient, which ranges from 0 perfect equality to 1 perfect inequality. All data for the socioeconomic factors were obtained from the American Community Survey 5-year (2012–2016) estimates.

#### Individual-level variables

##### Outcome.

The primary exposure variable is willingness to use PrEP, which was assessed via the question,

“If a pill (drug/medication) that could prevent transmission of HIV from an infected (HIV positive) partner to an uninfected (HIV negative) partner were available, l would take it.” Responses were yes, no, maybe. A binary variable was derived by collapsing the last two categories (yes vs. no/maybe).

##### Covariates.

The following individual sociodemographic covariates were included in the analysis: continuous age (in years); gender (male vs. female), educational attainment (less than high school, high school graduate or GED, some college, college degree or higher).

##### Other variables of interest

Awareness of PrEP was assessed via the question, “There is a pill (drug/medication) that you can get from your doctor daily to prevent transmission of HIV from an infected (HIV positive) sex partner to an uninfected (HIV negative) partner.” A new binary variable was derived by collapsing the last two categories (true vs. false/don't know).

Individual HIV risk was an index variable operationalized as a binary variable. Participants were considered to be at high HIV risk, if they met the following criteria within the last 3 months prior to survey administration: more than one sexual partner (anal or vaginal), more than one sexual partner and no condom use, or diagnosis with a sexually transmitted infection (STI) (gonorrhea, chlamydia, herpes, syphilis, trichomoniasis, genital warts, human papilloma virus or HPV). Participants reporting male-to-male sexual behavior, lifetime illicit drug use (e.g., powder or crack cocaine, heroin, or crystal methamphetamine), transactional sexual behavior, or identifying as a male-to-female transgender individual were also considered high-risk.

### Statistical analysis

#### Descriptives

We developed a geographic map of the distribution of 2016 NSHBC participants by ZIP code, and we included a map of Black people living with HIV in 2015, which we obtained directly from AIDSVu [37]. We included a figure with the distribution of the cities representing the ZIP codes from the new HIV diagnoses data that were matched to the study sample participants in the 2016 NSHBC.

We then described the individual-level covariates, variables of interest, and PrEP willingness using survey-weighted means for continuous variables and percentages for categorical or binary variables. We conducted bivariate statistical tests to compare individuals in the analytic sample (*n* = 169) to those who also lived in the South but resided in ZIP codes that could not be linked to the new HIV diagnosis data (*n* = 268). We then tested an interaction model for excluded versus included participants with the individual-level variables. We presented the results of those models along with the descriptive results. For the area-level variables, we conducted Spearman correlations.

#### Multivariable regressions

We used log-binomial regression within the generalized linear model (GLM) to approximate the prevalence ratio (PR) for the binary PrEP willingness outcome [48]. Statistical significance was evaluated through 95% confidence intervals. A multilevel model was not necessary because there was little to no clustering of individuals within ZIP codes (i.e., almost 1:1 match), which did not violate the independence assumption. We conducted two models. The first model assessed the likelihood of individual PrEP willingness as a function of area-level HIV risk and the following variables and covariates: age, gender, education, individual HIV risk, and awareness of PrEP (model 1). The second model assessed whether area-level socioeconomic factors attenuated the association between area-level HIV risk and PrEP willingness. We therefore added to model 1, area-level SED and income inequality (Model 2).

All area-level variables have been standardized to have a mean of 0 and standard deviation of 1. We considered nonlinear forms of the socioeconomic factors (e.g., categorical distributions), but results were not different when we used the continuous versions. Lastly, we also considered the proportion of persons uninsured in the ZIP code, but that covariate was not significant and did not alter the associations between the socioeconomic factors on the PrEP willingness outcome. We therefore kept the continuous versions of the socioeconomic factors and excluded the insurance variable to save degrees of freedom and have a parsimonious model. We analyzed the data using Stata 15.0 software [49] using the suite of “svy” commands to account for the weighted design of the NSHBC.

## Results

### Descriptives

**Fig. 1.**
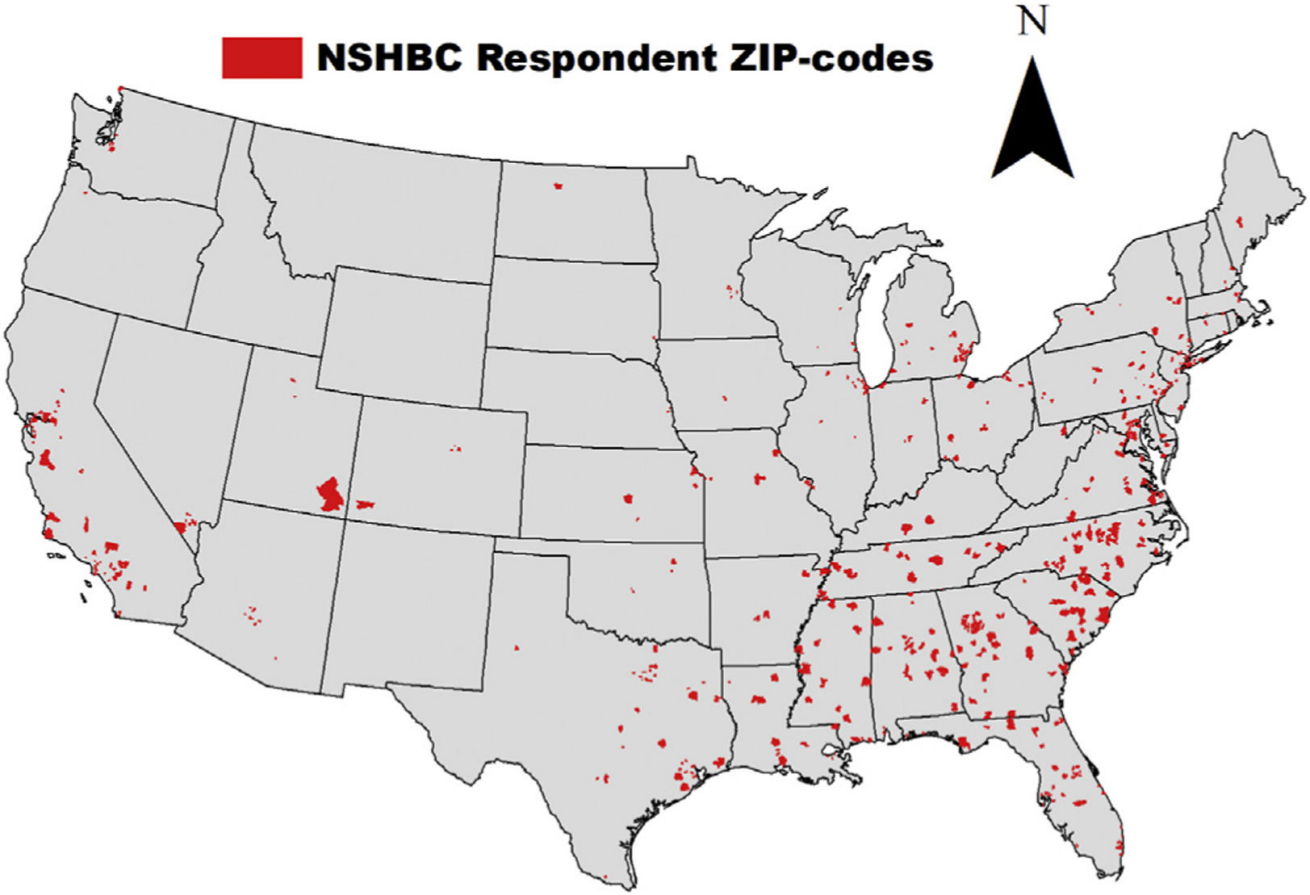
Distribution of NSHBC, 2016 participants across the United States.

**Fig. 2.**
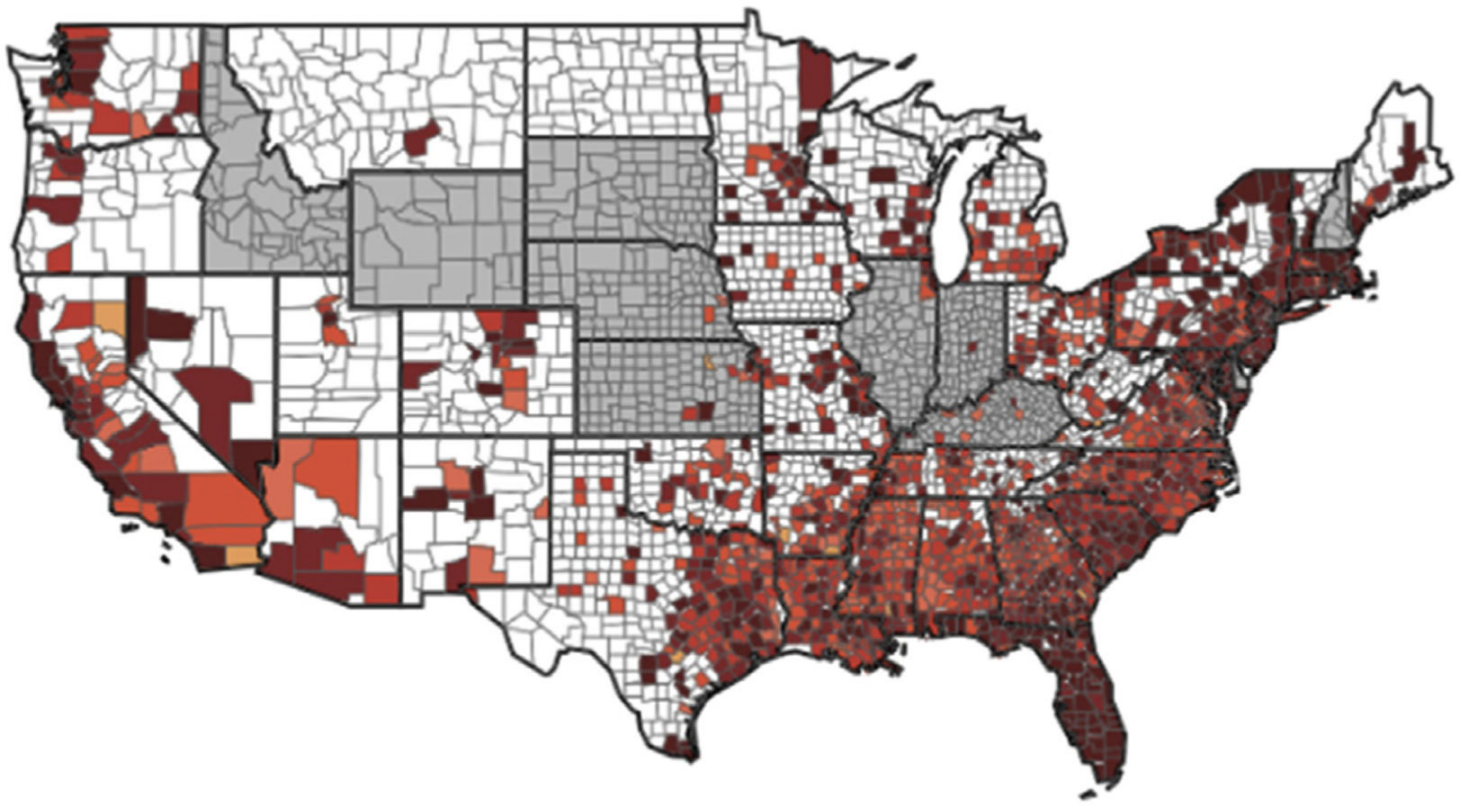
Rates of Black people living with HIV in the United States, 2015 (from AIDSVu).

**Fig. 3.**
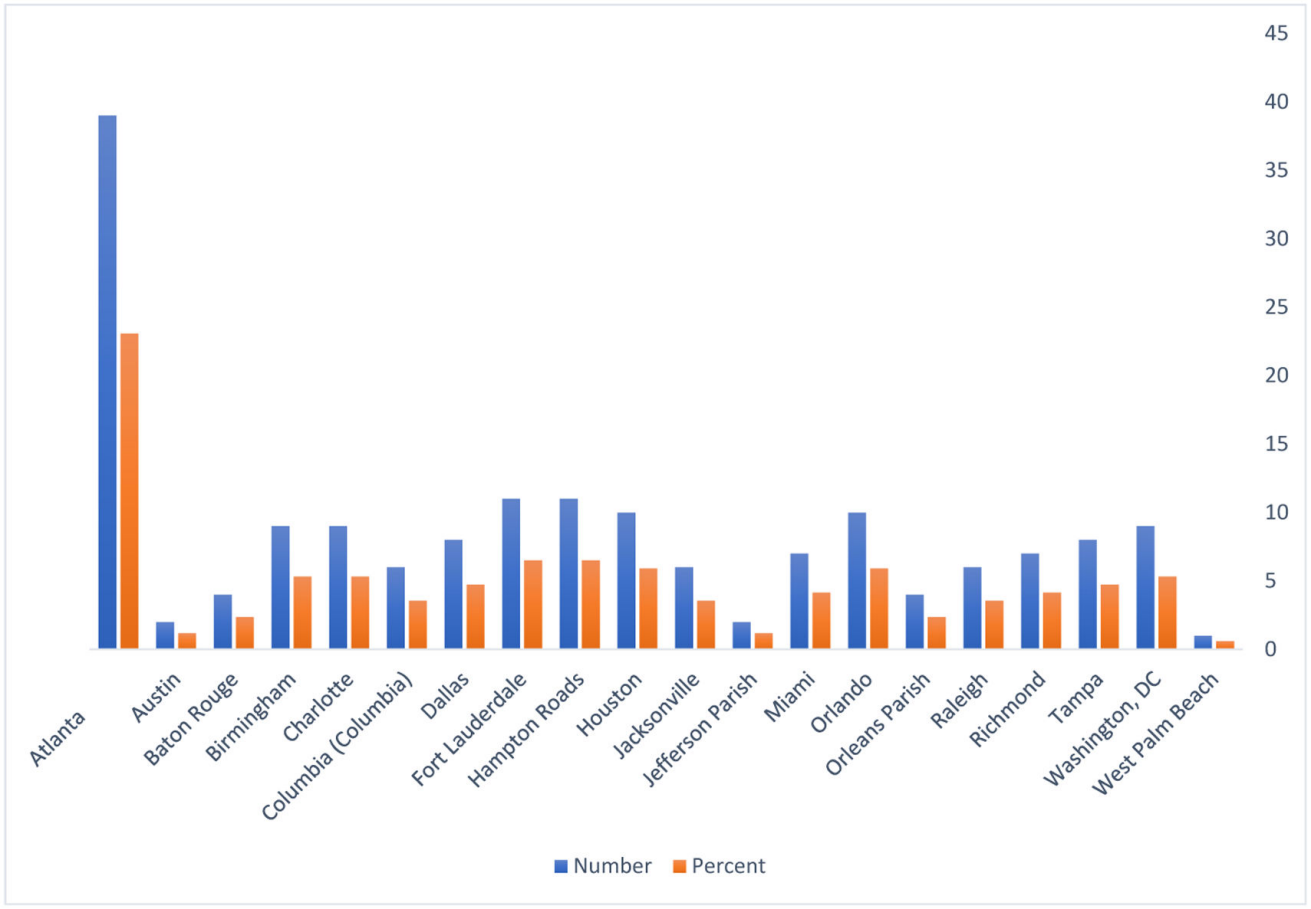
Cities that had ZIP-code data on new HIV diagnosis from AIDSVu that matched participants’ ZIP codes in the NSHBC, 2016.

Figure 1 shows the geography of the majority (i.e., >50%) of NSHBC participants residing within ZIP codes in the South. Figure 2 shows that the highest number of Black people living with HIV are in the South. Figure 3 shows the distribution of the cities from which we linked ZIP codes to participants in NSHBC. Twenty-three percent of participants resided in ZIP codes from Atlanta, GA, and six-and-a-half percent were from Fort Lauderdale, FL, and Hampton Roads, VA.

Table 1 displays the distribution of the variables in the study and compares the analytic sample to the remainder of the South sample that were excluded for reasons we described earlier. On average, participants in this study were 3 years older (*P* = .01) and more likely to be female (*P* = .05). There were no statistically significant differences in education, HIV risk, PrEP awareness, and PrEP willingness between participants included and those excluded. In the multivariable interaction model, there were no significant differences in the magnitude of association between age, sex, education, and awareness on willingness to use PrEP based on whether a participant was excluded or included. However, the association between HIV risk on willingness to use PrEP was significant with twice higher likelihood among individuals excluded (aPR = 2.56, 95% CI = 1.69, 3.90) compared to those included (aPR = 0.95, 95% CI = 0.43, 2.10).

Table 2 displays Spearman correlations for the area-level variables in the study. ZIP codes with increases in new HIV diagnosis between 2014 and 2015 were significantly and positively correlated with all socioeconomic factors. For instance, the correlation with percentage of persons below the Federal poverty level was 0.49, *P* = .00. Income inequality was positively correlated with poverty, which is an indication of the validity of these measures. Next, median household income was highly correlated with poverty (rho = 0.89), which provides support for our decision to create a SED index to reduce multicollinearity in the regression models.

### Multivariable regressions

Table 3 displays the results from the multivariable regressions. In model 1, we estimated the association between area-level HIV risk and willingness to use PrEP among participants. We found that participants living in ZIP codes within the upper 25th percentile of changes (increases) in new HIV diagnosis were twice as likely to be willing to use PrEP (aPR = 2.03, 95% CI = 1.03–4.02), *P* = .03 compared to participants living in ZIP codes within the bottom 75th percentile. In this regression, the individual-level variables were not statistically significant.

In model 2, we examined whether accounting for area-level socioeconomic factors would attenuate the association between area-level HIV risk and PrEP willingness. We found that adjusting for SED and income inequality attenuated that association to such a degree that area-level HIV risk was no longer significant (aPR = 1.63, 95% CI = 0.78–3.39, *P* = .19). Post-hoc calculations showed a 20% difference in the coefficients of area-level HIV risk before and after adjustment of the socioeconomic factors (i.e., [{2.03–1.63}/2.03] * 100). None of the socioeconomic factors, however, were statistically significant.

## Discussion

The burden of new HIV infections and lifetime risk of acquiring HIV in the United States are highest among Black people [50] and in the U.S. South [51, 52]. More than 50% of Black people in the United States live in the South [53]. Therefore, the nexus of race/ethnic and geographic risk of HIV [13] makes studying HIV among Black people in the South paramount to reduce disparities in HIV. Furthermore, we highlighted that while there are numerous individual factors that potentiate HIV risk among Black people, there are several area-level factors (e.g., community-level indicators of transmission such as HIV incidence) [27] that have received limited attention. We therefore investigated the association between area-level HIV risk and willingness to use PrEP among Black people living in the U.S. South.

The first major finding that advances knowledge regarding predictors of PrEP uptake is that area-level HIV risk, *not* individual-level HIV risk, was significantly associated with higher willingness to use PrEP, when both factors were considered simultaneously. Based on Diffusions of Innovations Theory applied to HIV, one potential intervention could be to increase the exposure to PrEP advertisements in specific media markers with high HIV prevalence. Mass media campaigns, for instance, the “truth” campaign was shown to successfully reduce teenage smoking prevalence [54], and perhaps other culturally appropriate [55] campaigns could be developed for PrEP engagement among Black people in high-HIV prevalence settings [56, 57]. Another implication of our finding the time opportunity to shape prescribing practices. The current discourse about increasing uptake of PrEP is to educate providers to identify individual risk factors of PrEP uptake (e.g., injection drug use or condomless sex) [58] and weigh potential benefits compared to their perceived biases or racial stereotypes [59]. For instance, one study identified that medical school students—future health-care providers—were more likely to believe that Black compared to White patients would engage in risk compensation (increased unprotected sex if prescribed PrEP) [21]. Perhaps, this study can motivate a new conversation to encourage providers to more closely consider the guidelines regarding those “in high HIV prevalence area or network,” and start measuring the extent to which clinicians prescribe PrEP based on that criterion.

The study findings of nonsignificant associations between individual-level HIV risk and PrEP willingness add to a body of existing evidence that individual behavioral factors alone are insufficient to account for increased HIV risk among Black people [32, 60] and, we add, may not be sufficient to explain low uptake of PrEP. Our study indicates a potential role for environment to enter physician–patient conversations when discussing eligibility for PrEP. Although limited time is a critical factor in medical visits, there are emerging efforts to make area-level social determinants of health readily available for physicians to inform a more comprehensive assessment of risk [61]. For instance, NowPow's HealtheRx and CommunityRx are systems that curate area-level resources within a patient's medical chart so that physicians can better understand their patients’ risk environment but also potential resources available [62]. These examples may represent possible avenues to incorporate area-level factors into prescribing PrEP.

The second major contribution of our paper is that socioeconomic factors attenuate some of the positive association between area-level HIV risk and individuals’ willingness to use PrEP. Specifically, after adjusting for socioeconomic deprivation and income inequality, the association between area-level HIV risk and willingness to use PrEP diminished by 20% and was no longer significant. Although the socioeconomic factors were not significant, potentially because of low power to detect variation, these overall findings validate the negative impact of socioeconomic disadvantage. Specific to the U.S. South (compared to other regions); social, economic, and political factors such as higher levels of poverty, lack of insurance access, longer distances to travel for care, have been identified as key facilitators of HIV risk [63, 64].

In our study, the proportion of uninsured people was not a significant predictor and did not explain any additional reduction (on top of the socioeconomic factors) in the association between area-level HIV risk and willingness to use PrEP. While other research shows a protective association between public spending on social services per person in poverty and HIV case rates [65], our study findings potentially suggest that economic factors could be a priority to address in the context of limited affordable care coverage in the South.

There are some limitations of this study. While our research question was novel, our sample of 169 participants (38.7% of the South sample) who could be linked to ZIP codes with new HIV diagnosis data is small. There are several reasons why ZIP codes could not be linked, although we cannot know for certain based on the information provided by AIDSvu. Among the excluded, there could be ZIP codes with few cases (e.g., <5), which would be and concealed from the general public due to reliability issues and privacy. This limitation is likely to affect studies seeking to replicate this analysis in rural areas in the South. For our study, however, many of the cities were in urban populated areas (e.g., Houston, TX, and Fort Lauderdale, FL). Second, data could have been unavailable for a particular ZIP code because AIDSVu, the external data set used in this analysis, was not able to get such data from the public health agency serving that particular ZIP code. However, we are unable to distinguish the actual reason that a particular ZIP code was missing HIV data.

Participants in the sample did not differ from those excluded based on education, individual HIV risk, PrEP awareness, or PrEP willingness. In a multivariable model, we did find higher association between HIV risk and PrEP willingness among those included. With a larger sample size, future studies should examine a potential interaction between area-level HIV risk and individual-level HIV risk on PrEP.

Sampling bias could have influenced our results because surveys were conducted from a Web panel of respondents that may not be fully representative of the population in the cities under study. Nevertheless, the weighting methodology of the NSHBC ensures that the distribution of sample participants matches the distribution of the U.S. adult population along key dimensions such as age, gender, race/ethnicity, census region, household income, home ownership, and residence in or out of a metropolitan statistical area [44]. Additionally, Census 2010 data show that, among people who report Black race, the highest proportion is distributed across three southern states: Florida, Texas, and Georgia [53]. In our study, the highest proportion of ZIP codes among participants were in those same states (e.g., Atlanta, GA, Dallas and Houston, TX, and Fort Lauderdale, Jacksonville, Orlando, and Tampa, FL). Therefore, even with this small sample, given the weighting and the sample distribution reflecting true population distributions, our results are potentially generalizable to the U.S. adult Black population in the South.

Future studies with larger samples of individuals across other geographic scales (e.g., Census tracts) are necessary. We did not include spatial clustering effects because there were a few cities and the data were not from contiguous counties (for e.g., Dallas to Atlanta is approximately 11 hours’ drive via I-20 E). This approach was unnecessary and as mentioned, multilevel models were not appropriate either.

There may have been other unobserved area-level confounders influencing our results. For instance, crime indicators such as assault rate or crime index are known predictor of HIV incidence [29] and late HIV diagnosis [66]. People who live in areas with higher crime may be preoccupied with survival and associated psychological threats, such that self-perceived HIV risk and willingness to use medication are of lower priority [67]. Moreover, income inequality—one of the variables in this study—is a risk factor for crime as scare resources may cause people to use illegal means to survive [68]. Social cohesion and capital are overlapping SDOH constructs, which are associated with willingness to use PrEP [69]. Social cohesion is a property of groups or communities. Information exchange within communities is one mechanism proposed to link social cohesion to HIV testing and other HIV prevention behavior. Therefore, the level of information exchange in the area (particularly around HIV prevalence) could potentially explain some of the variation in willingness to use PrEP. However, social cohesion data at the ZIP-code level were not available.

We did not examine the potential for a cross-level interaction between area-level HIV risk and individual-HIV risk. Specifically, it is possible that individuals who engage in high HIV risk behaviors and live in areas with high HIV risk may have a stronger likelihood of willingness to use PrEP if they are more socially conscious of their behavior and their risk environment. Therefore, including only main effects for each level of this exposure could potentially miss important dynamics between participants and their environment [70]. Nevertheless, future analyses should consider the potential effect modifications when specifying multivariable models for PrEP willingness and PrEP uptake, when larger samples are available.

We did not stratify the data between individuals who report engaging in male-to-male sex compared to individuals who report heterosexual sex. We know that HIV risk is highest among Black MSM and rates of HIV among this group are highest in the U.S. South [71]. However, we did not have the sample size to stratify the analysis. Nevertheless, male-to-male sex was one criterion in the HIV risk composite variable, which we included as a variable of interest in the models, and this composite variable was not significant.

Despite these limitations, we examined a topic that has received limited attention in the research thus far. We used a nationally represented sample and conducted weighted analyses, which give us some confidence that these results are potentially generalizable to the larger population of Black people in the U.S. South. We utilize population-level external data on new HIV diagnoses from AIDSVu and socioeconomic factors from the Census Bureau and linked these data to participants’ ZIP code of residence. Our methodology could easily be replicated by researchers who are interested in moving beyond individual-level explanations for HIV prevention among Black people.

## Conclusions

We present area-level HIV risk as a factor that potentially influences willingness to use PrEP among individuals. We recommend replicating this study using larger sample size and potentially other geographic areal units (e.g., Census tracts). Future work on this topic should also investigate other potential area-level measures of HIV risk (e.g., prevalence of HIV mistrust or incarceration rates) as well as area-level and individual-level mechanisms and pathways that influence individuals' decisions. Investigating incarceration rates within areas could have a strong impact on community norms that breed mistrust in government systems including health care. Area-level mistrust can then influence individual's perceptions of why the “system” is proposing PrEP and stymie willingness to use this technology. Other potential avenues for this work include measuring an individual's awareness of HIV prevalence and places that prescribe PrEP in their neighborhood along with indicators of PrEP marketing and other area-level cultural variables such as racial discrimination prevalence.

## Figures and Tables

**Table 1:** Selected characteristics of Black people in the U.S. South participating in the NSHBC, 2016

N (weighted col %) or weighted mean (SD.) Prevalence ratio (PR) and (95% CI)	Analytic sample (*n* = 169)	Excluded sample* (*n* = 268)	*P*-value^‡^
Age	36.3 (8.58) PR = 1.01 (0.97, 1.05)	33.3 (0.07) PR = 1.01 (0.99, 1.03)	.01
Gender			
Female, ref	114 (62)	110 (50)	.05
Male	55 (38) PR = 1.00, (0.44, 2.26)	158 (50) PR = 0.69, (0.45, 1.05)	
Education			
Less than high school, ref	11 (12)	18 (11)	.52
High school diploma or GED	33 (28) PR = 0.65, (0.18,2.34)	63 (36) PR = 0.48, (0.22, 1.04)	
Some college	62 (35) PR = 0.80, (0.25, 2.59)	101 (34) PR = 0.87, (0.47, 1.62)	
College degree or higher	63 (25) PR = 0.50, (0.16, 1.60)	86 (19) PR = 0.53, (0.26, 1.09)	
HIV Risk^†^(Yes)	44 (26) PR = 0.95 (0.43, 2.10)	58 (20) PR = 2.56 (1.69, 3.90)^§^	
Aware of PrEP (Yes vs. No/Unsure)			
Yes	26 (16) PR = 1.35, (0.57, 3.21)	44 (15) PR = 1.15, (0.68, 1.95)	.72
Willing to use PrEP (Yes vs. No/Unsure)			
Yes	40 (25)	75 (27)	.60

* Sample who lived in the U.S. South but resided in ZIP codes where we did not have HIV data from AIDSVu.org.

† Individual HIV risk was an index variable where those meeting criteria had: (more than one sexual partner in the last 3 months; or more than one sexual partner and no condom use in the last 3 months; or more than one sexual partner, anal sex and no condom use in the last 3 months; and/or were diagnosed with a sexually transmitted infection (STI) (gonorrhea, chlamydia, herpes, syphilis, trichomoniasis, genital warts, human papilloma virus or HPV) in the 3 months prior to the survey; and/or male-male sexual behavior; and/or transgender (M to F); and/or illicit drug use [e.g., powder or crack cocaine, heroin, or crystal meth] use in a lifetime; or any transactional sexual behavior).

‡ *P*-value is comparing the difference in the mean or proportion of the excluded versus included sample.

§ Statistically significant interaction, Chi-square 1 (855), F = 4.71, *P* = .03.

**Table 2:** Spearman rho correlations: ZIP-code level factors among 196 Black people that resided in 142 unique ZIP codes at the time of the study, NSHBC, 2016

	1	2	3	4	5
1. Area-level HIV risk (Change in HIV diagnosis rates, 2014 to 2015)	1				
2. Percentage below the Federal poverty level	0.49	1			
3. Percent unemployed	0.30	0.68	1		
4. Median household income	0.41	0.89	0.68	1	
5. Income inequality (GINI coefficient)	0.35	0.48	0.16*	0.41	1

All *P*-values were less than .01 unless indicated.

* *P*-value = .04.

**Table 3:** Results from regression analysis of ZIP-code level factors in association with willingness to use PrEP among Black people in the U.S. South, NSHBC, 2016.*n* = 196 participants in 142 unique ZIP codes

	Model 1	Model 2
	Prevalence ratio (95% confidence interval), *P*-value	Prevalence ratio (95% confidence interval), *P*-value
Individual-level variables		
Age	1.00 (0.97, 1.04), *P* = .82	1.00 (0.97, 1.04), *P* = .92
Female (ref = male)	1.13 (0.46, 2.78), *P* = .78	1.06 (0.44, 2.54), *P* = .90
Education (ref = Less than high school)		
High school diploma or GED	0.73 (0.18, 2.87), *P* = .65	0.66 (0.18, 2.45), *P* = .54
Some College	1.01 (0.32, 3.18), *P* = .99	0.97 (0.31, 3.06), *P* = .96
College degree or higher	0.68 (0.22, 2.15), *P* = .52	0.68 (0.21, 2.17), *P* = .52
HIV risk, yes (ref = no)	0.92 (0.42, 2.04), *P* = .85	0.90 (0.43, 1.88), *P* = .77
Aware of PrEP, yes (ref = no)	1.46 (0.58, 3.70), *P* = .42	1.26 (0.49, 3.23), *P* = .63
Area-level variables		
HIV Risk (change in new HIV diagnosis rates, 2015–2014)		
Upper 25th percentile (ref = bottom 75th percentile)	2.03 (1.03, 4.02), *P* = .03	1.63 (0.78, 3.39), *P* = .19
Socioeconomic deprivation		1.08 (0.76, 1.52), *P* = .66
Income inequality (GINI coefficient)		1.32 (0.96, 1.82), *P* = .09

Area-level variables are z-scored to have a mean of 0 and SD of 1. Individual HIV risk is an index variable where those meeting criteria had: (more than one sexual partner in the last 3 months; or more than one sexual partner and no condom use in the last 3 months; or more than one sexual partner, anal sex and no condom use in the last 3 months; and/or were diagnosed with a sexually transmitted infection (STI) (gonorrhea, chlamydia, herpes, syphilis, trichomoniasis, genital warts, human papilloma virus or HPV) in the 3 months prior to the survey; and/or male-male sexual behavior; and/or transgender (M to F); and/or illicit drug use [e.g., powder or crack cocaine, heroin, or crystal meth] use in a lifetime; or any transactional sexual behavior). Socioeconomic Deprivation is an index of percentage below the federal poverty level, percent unemployed, median household income (Cronbach's alpha = 0.88).
